# Mechanistic Insight into Phenolic Compounds in Mitigating Diabetic Complications Induced by Advanced Glycation End Products

**DOI:** 10.3390/cimb47100841

**Published:** 2025-10-14

**Authors:** Wajid Zaman, Adnan Amin

**Affiliations:** Department of Life Sciences, Yeungnam University, Gyeongsan 38541, Republic of Korea; wajidzaman@yu.ac.kr

**Keywords:** diabetes, milliard reaction, microvascular complications, stress signaling, molecular dynamics

## Abstract

Diabetes mellitus is a chronic metabolic disorder that facilitates the formation of advanced glycation end products (AGEs), which contribute to oxidative stress, inflammation, and vascular damage, causing complications including nephropathy, neuropathy, and atherosclerosis. AGEs are primarily synthesized through the Maillard reaction, alongside various signaling pathways. Activation of the receptor for AGE (RAGE) triggers inflammatory signaling pathway cascades, exacerbating tissue damage. Phenolic compounds found in plant-based foods, such as quercetin and resveratrol, have shown promise in counteracting AGE-related complications through their antioxidant and anti-inflammatory effects that inhibit AGE formation, reduce oxidative stress, and modulate RAGE signaling, while also enhancing insulin sensitivity and improving glucose homeostasis. Indeed, quercetin can help prevent AGE accumulation and reduce diabetic nephropathy, while resveratrol activates the SIRT1 pathway, improving insulin sensitivity. This review examines the mechanisms through which phenolic compounds mitigate AGE-induced diabetic complications, using computational, in vitro, preclinical, and clinical evidence. This review also explores the synergistic effects of these compounds with conventional antidiabetic drugs, addresses bioavailability challenges, and suggests future research directions. Overall, this review offers a comprehensive understanding of the role of phenolic compounds in managing diabetes, underscoring their potential as complementary agents in diabetes therapy and developing more effective natural treatments.

## 1. Introduction

Diabetes mellitus (DM) represents a complex, multifactorial metabolic disorder that contributes significantly to the global burden of chronic disease, affecting millions worldwide [1]. The dysregulation of glucose homeostasis is central to the pathophysiology of DM, leading to chronic hyperglycemia [2]. Subsequently, persistently elevated glucose levels result in the accumulation of advanced glycation end products (AGEs), which are formed through the non-enzymatic glycation of proteins, lipids, and nucleic acids [3]. The formation of AGEs is accelerated under conditions of hyperglycemia, while the accumulation of AGEs plays a pivotal role in the development of various diabetic complications, such as nephropathy, retinopathy, neuropathy, and cardiovascular disease [4,5]. The biochemical mechanisms through which AGEs contribute to these complications are complex, involving interactions with cellular receptors, such as the receptor for advanced glycation end products (RAGEs), which trigger inflammatory responses, oxidative stress, and cellular dysfunction [6,7]. These cellular perturbations significantly impact tissue integrity, leading to the progression of diabetic complications across multiple organs, including the kidneys, retina, skin, and vasculature [8].

The impact of AGEs on cellular and molecular pathways is primarily mediated through their interaction with RAGEs, a multi-ligand receptor that initiates a cascade of signaling events upon binding to AGEs. Therefore, the AGE–RAGE axis is crucial in the induction of inflammatory cytokines, matrix metalloproteinases, and various other molecules that facilitate tissue damage and fibrosis, driving the progression of diabetic complications [9]. The activation of downstream signaling pathways, including the nuclear factor kappa-light-chain-enhancer of activated B cells (NF-κB), mitogen-activated protein kinase (MAPK), and phosphoinositide 3-kinase (PI3K)/Akt, leads to the amplification of oxidative stress [10], inflammatory responses, and cellular apoptosis, further contributing to the deterioration of cellular function [11]. Additionally, the deposition of AGEs in tissues causes structural modifications in the extracellular matrix, impairing tissue function and promoting the pathological remodeling observed in diabetic complications [12]. Thus, understanding these molecular mechanisms is crucial for developing therapeutic strategies that aim to prevent or reverse AGE-induced cellular damage, particularly in the context of diabetes management. Polyphenolic compounds, which are abundant in plant-based foods, have attracted significant attention for their potential to mitigate the deleterious effects of AGEs [13]. These compounds are best known for possessing potent antioxidant and anti-inflammatory properties, which enable the compounds to scavenge free radicals and reduce oxidative stress, a process that plays a central role in AGE formation [14]. Polyphenolic compounds are characterized by more than one hydroxyl group attached to an aromatic ring [15]. Some broad polyphenolic compound classes include Flavonoids (quercetin, kaempferol, rutin), phenolic acids (allic acid, chlorogenic acid), stilbenes (resveratrol), lignans (secoisolariciresinol) and Tannins (proanthocyanidins) [16]. Certainly, each compound class has unique structural feature that mainly contribute towards biological activity for instance, in the case of flavonoids, the “flavonoid backbone” that possesses ring A and B, connected by a three-carbon bridge (C ring) is of prime importance [17]. Further, the positioning of OH groups and degree of methylation, hydroxylation and glycosylation also play a vital role in biological activity [18]. In case of phenolic acid, the carboxylic acid group (-COOH) and vinyl group (–CH=CH2) attached to the aromatic ring are of prime importance with respect to biological activities [19]. Similarly, in stilbenes the “stilbene backbone’ and in Lignans, the existence of two phenylpropanoid units that are linked by a central carbon–carbon bond is important. Lastly, in case of Tannins, the occurrence of multiple phenolic groups allows them to interact with various biological targets, including AGEs [20].

The polyphenolic compounds can directly inhibit the Maillard reaction, the process responsible for AGE formation, thus preventing the accumulation of these harmful compounds [21]. In addition to their role in reducing AGE formation, polyphenols also modulate the AGE–RAGE signaling pathway, attenuating the downstream inflammatory responses and oxidative stress induced by AGE accumulation [22]. This dual action, which reduces AGE formation and mitigates AGE-mediated cellular damage, highlights the therapeutic potential of polyphenolic compounds in managing diabetic complications. Several studies have demonstrated that dietary polyphenols, such as flavonoids, phenolic acids and stilbenes, possess the ability to regulate glycemic control, reduce AGE levels, and protect against diabetic nephropathy (DN), retinopathy, and cardiovascular disease [23,24,25]. Further the Anti-AGEs potential of polyphenolic compounds in prediabetic conditions is also important to consider [26]. In a recent investigation, the polyphenolic compounds were analyzed in prediabetic conditions and outstanding outcomes were recorded that show potential of polyphenolic compounds against AGEs [27,].

However, the bioavailability of polyphenolic compounds remains a critical factor in their therapeutic efficacy. Despite the promising biological activities of these polyphenolic compounds, the absorption and metabolism of polyphenols are often limited by factors such as low solubility, rapid metabolism, and poor systemic availability [28]. Hence, significant efforts have been made to enhance the bioavailability of these compounds through various biotechnological approaches. These include the development of advanced drug delivery systems, such as nanoparticles and liposomes, which improve the stability, solubility, and absorption of polyphenols. Furthermore, strategies aimed at improving the pharmacokinetics of polyphenolic compounds, such as modifying the chemical structure or inducing combinations with other bioactive agents, have been explored to maximize the therapeutic potential of these compounds in diabetes management [29,30].

This review aims to provide a comprehensive overview of the pathogenesis of AGEs in diabetes and the role of polyphenolic compounds in modulating AGE formation and mitigating diabetic complications. The scope of the review encompasses a detailed examination of the molecular mechanisms through which AGEs contribute to the development of diabetes-related complications, with particular emphasis on the AGE–RAGE signaling pathway and the subsequent downstream effects. This review also highlights the current state of research and future directions for studying polyphenolic compounds as potential therapeutic agents in the prevention and treatment of diabetic complications associated with AGEs.

## 2. Literature Search and Methodology

The review is built on a comprehensive investigation of several peer-reviewed journal articles, review papers and book sources that comprised information, findings related to AGEs and the effect of plant polyphenolic compounds in reducing their effects. Several academic databases for instance, Web of Science, ScienceDirect, Wiley, MDPI, Google Scholar and Springer were retrieved for data. The required information was obtained by certain key words including “Diabetes Mellitus”, “Advance glycation”, “Diabetic complications”, “plant polyphenols” “Mechanistic of AGEs inhibition by phenolics”, “AGE-RAGE Signaling pathways”, “AGE-RAGE computational tools”, “ Network pharmacology of phenolics in AGEs” and “Challenges in AGEs research” Use of Boolean operators “AND”, “OR” was accomplished to improve quests and have a more detailed overview. The timeframe for included publications primarily ranged from 1995 through 2025 to include introductory research papers as well as the latest and modern developments in AGEs. The set “inclusion criteria” was delegated to include studies about Diabetes, AGEs and polyphenolic compounds only. All included articles were limited to peer-reviewed works published in the English language only. The “exclusion” criteria were set that limited the use of conference abstracts, literature published before the year 1995, unpublished data, and retracted literature to maintain academic rigor. Initially, a data set of approximately 370 scientific records was saved and curated by “title” and “abstract” for relevance and eminence. Following this, 310 articles were reviewed in full text, and only 280 articles were able to meet the criteria for inclusion in the project. This method warranted a wide but very intensive exposure of the topic. All figures included in this review article were generated by the authors using BioRender.com for more detailed and conceptual illustrations. All information was sensibly vetted to confirm a precise depiction of the discussed content (Table 1).

## 3. Diabetic Complications and AGEs

AGEs are considered critical in the pathogenesis of diabetes and its allied serious complications. The formation of AGEs is closely linked to hyperglycemia, and these compounds accumulate over time, contributing to the long-term complications observed in diabetic patients [7,31]. AGE accumulation is particularly problematic in tissues with long turnover rates, such as the skin, kidneys, retina, and vascular systems, where the AGE can exacerbate the damage caused by diabetes [32].

### 3.1. AGE Formation Mechanism and the Mechanistic Role in Diabetic Complications

AGEs form primarily through the Maillard reaction, where reducing sugars bind to the free amino groups of proteins, creating an initial reversible product [33], which eventually undergoes further chemical modifications to form irreversible AGEs [22]. The non-enzymatic glycation of proteins, lipids, and nucleic acids promotes the formation of cross-linked structures, which accumulate in various tissues over time [34]. Under hyperglycemic conditions, the rate of AGE formation is significantly accelerated, resulting in the accumulation of AGEs in tissues such as the skin, kidneys, eyes, and blood vessels [5]. These accumulated AGEs modify the structure and function of macromolecules, such as collagen and elastin, which are crucial for maintaining tissue elasticity and function [35]. Additionally, AGEs interact with specific receptors, most notably the RAGE, leading to the activation of proinflammatory pathways and an increase in oxidative stress [36]. This results in a cascade of molecular events that contribute to the development of diabetic complications, including vascular stiffening, renal damage, and retinal degeneration.

The formation process for AGEs starts with the development of a Schiff base between the carbonyl group of the sugar and the amino group in the target molecule. This Schiff base is rearranged to form an Amadori product, which represents a more stable but still reactive intermediate [37]. Over time, Amadori products undergo further chemical modifications, including oxidation, dehydration, and cross-linking, resulting in the formation of AGEs [38]. These reactions are accelerated in the presence of hyperglycemia, oxidative stress, and chronic inflammation, which are prevalent in diseases such as diabetes and aging [39].

### 3.2. Major AGE Types

**Figure 1:**
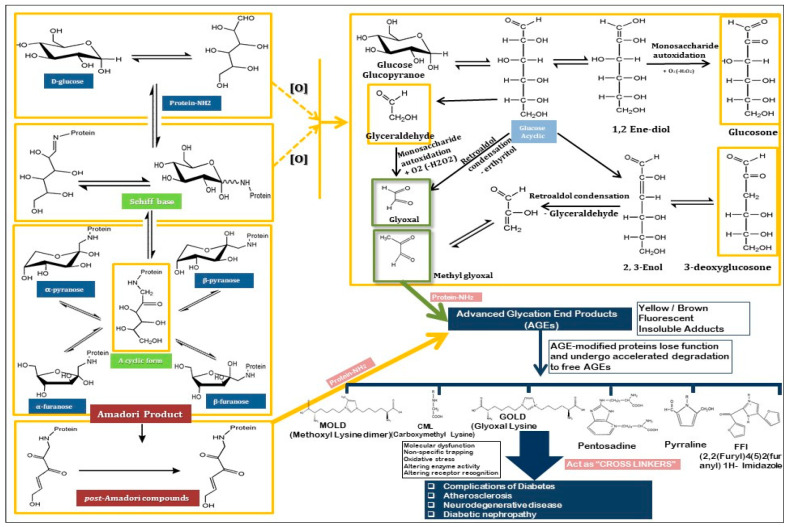
The mechanism of Maillard reaction through which AGEs are formed, the and the presentation of various AGEs.

Methylglyoxal (MGO) and other reactive dicarbonyl compounds, such as Nε-carboxymethyllysine (CML) and pentosidine, represent well-known AGEs and are highly reactive intermediates in the glycation pathway. Additionally, several other types of AGEs are recognized for their roles in various diseases [40]. MGO-derived hydroimidazolone (MGO-H1) and glyoxal-derived hydroimidazolone (GH1) are significant AGEs formed from an interaction between MGO and glyoxal with protein residues [41]. Other notable AGEs include pyrraline, a cross-linking AGE formed by the reaction between pentoses that contain a lysine and 3-deoxyglucosone (3-DG), an intermediate that can extensively modify proteins. Ne-(carboxyethyl)valine (CEV) is another important AGE, and arises from the modification of valine residues by reactive carbonyl species [42]. Additionally, the glyoxal-lysine dimer (GOLD) and MGO-lysine dimer (MOLD) are AGEs formed by the reaction between glyoxal and MGO with lysine residues, respectively (Figure 1). These dimeric AGEs have been implicated in the cross-linking of extracellular matrix proteins, contributing to tissue stiffness [43]. These AGEs, along with others such as the imidazolone family, play critical roles in the aging process and are associated with chronic diseases, including cardiovascular disorders, neurodegeneration, and diabetes.

### 3.3. Pathophysiological Consequences of AGE-Induced Diabetic Complications

As explained earlier, an increased accumulation of AGEs in various tissues results in profound alterations in tissue architecture and function, contributing to the pathophysiology of several diabetic complications. AGEs in the kidneys promote the formation of advanced cross-links in collagen and elastin fibers, leading to the thickening of the glomerular basement membrane and mesangial expansion [44]. These changes compromise kidney function, impairing filtration and promoting the development of DN, which can lead to the onset of proteinuria. The increased production of proinflammatory cytokines and growth factors, such as TGF-β, further exacerbates the fibrosis and inflammation in renal tissue, contributing to the progressive nature of the disease. In diabetic retinopathy, AGE accumulation in the retinal vasculature leads to endothelial cell dysfunction, increased vascular permeability, and the formation of microaneurysms [45]. The leakage of fluid from these damaged vessels results in retinal edema, a key feature of diabetic macular edema, which can severely impair vision if left untreated.

**Figure 2:**
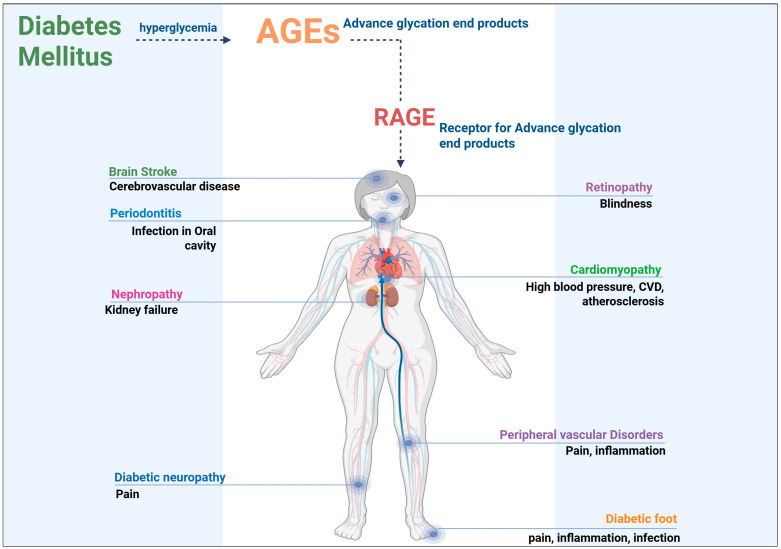
Pathological considerations of AGEs in diabetic patients.

Cardiovascular complications related to AGEs are primarily driven by the accumulation of these products in the vascular endothelium and smooth muscle cells, where the AGEs contribute to endothelial dysfunction, inflammation, and the promotion of vascular calcification. AGE-induced modifications of collagen and elastin in the vascular walls cause the arteries to stiffen, which increases blood pressure and contributes to the development of atherosclerosis [46]. These alterations also promote platelet aggregation, increasing the risk of thrombosis and myocardial infarction in diabetic patients [47]. Additionally, AGEs have been shown to activate the renin–angiotensin system, further exacerbating blood pressure regulation and contributing to the vascular complications observed in diabetes [48]. The cumulative effect of AGE-induced modifications in multiple organ systems significantly impairs overall health and accelerates the onset of severe complications, emphasizing the importance of understanding the role of AGEs in diabetes (Figure 2).

## 4. Signaling Pathways Involved in AGE-Induced Diabetic Complications

The pathophysiology of diabetic complications is significantly influenced by the accumulation of AGEs, which, in turn, trigger multiple intracellular signaling pathways that mediate tissue damage and dysfunction [49]. One of the key mechanisms through which AGEs exert their deleterious effects is through an interaction with the RAGE. A detailed discussion regarding important signaling pathways is presented below.

### 4.1. AGE–RAGE Signaling Pathway

The receptor for advanced glycation end products (RAGE) plays a central role in mediating the effects of AGEs on cellular function [50]. RAGE is a multi-ligand receptor that binds AGEs and triggers a series of intracellular signaling events that contribute to the pathological consequences of AGE accumulation [51]. Upon binding to AGEs, RAGE activates several downstream signaling pathways, including NF-κB, MAPKs, and the PI3K/Akt pathway, all of which promote inflammation, oxidative stress, and cellular dysfunction [52]. The activation of NF-κB, a key regulator of immune responses, leads to the production of proinflammatory cytokines such as TNF-α, IL-6, and IL-1β, which further exacerbate tissue inflammation and contribute to the development of diabetic complications [51]. Additionally, RAGE activation increases the production of reactive oxygen species (ROS), leading to oxidative stress, which accelerates cellular damage and promotes fibrosis in affected tissues [51].

The AGE-RAGE interaction also contributes to the development of insulin resistance, a key symbol of type 2 diabetes, by disrupting insulin signaling pathways and impairing glucose uptake in peripheral tissues such as muscle and adipose tissue [53]. In the vasculature, RAGE activation induces the expression of matrix metalloproteinases (MMPs), enzymes that degrade extracellular matrix components contributing to vascular remodeling and endothelial dysfunction [47]. Likewise in the kidneys, RAGE-mediated signaling promotes the accumulation of extracellular matrix proteins, contributing to glomerulosclerosis and tubulointerstitial fibrosis [54]. The interaction between AGEs and RAGE is thus central to the cellular dysfunction observed in diabetic complications, and therapeutic strategies aimed at modulating this interaction hold promise for alleviating AGE-induced damage.

### 4.2. AGE-Induced Activation of MAPK Pathways

MAPKs, including the extracellular signal-regulated kinase (ERK), c-Jun N-terminal kinase (JNK), and p38 MAPK, are key mediators of AGE-induced cellular dysfunction [55] and are involved in regulating various cellular processes, such as inflammation, apoptosis, and fibrosis [56]. In the context of diabetes, these kinases are activated downstream of the AGE–RAGE axis and contribute to the development of diabetic complications by promoting inflammatory responses and tissue remodeling [57]. The activation of ERK, JNK, and p38 MAPK in response to AGE exposure leads to the phosphorylation of several target proteins, including transcription factors and enzymes involved in cell survival, proliferation, and inflammation [58].

In particular, ERK activation promotes the production of proinflammatory cytokines and enhances cell survival in response to stress [59]. Conversely, JNK and p38 MAPK activation are primarily involved in regulating apoptosis and inflammation [60]. The activation of JNK and p38 MAPK in endothelial cells contributes to endothelial dysfunction and increased vascular permeability, which are key features of diabetic retinopathy and nephropathy. Moreover, p38 MAPK activation induces the production of profibrotic factors, such as TGF-β, which drives fibrosis in tissues, including the kidneys and heart [61]. MAPK-related signaling pathways are also involved in modulating insulin signaling, while the activation of these pathways can also impair insulin sensitivity, leading to insulin resistance in skeletal muscle and adipose tissue [62]. Thus, the AGE-induced activation of MAPK pathways plays a crucial role in driving inflammation, fibrosis, and insulin resistance in various tissues, contributing to the progression of diabetic complications.

### 4.3. NF-κB Pathway in AGE-Mediated Inflammation

The NF-κB pathway is a key regulator of the inflammatory response and is critically involved in the pathogenesis of diabetic complications induced by AGE accumulation [63]. NF-κB is a transcription factor that, upon activation, translocates to the nucleus and regulates the expression of genes involved in inflammation, immune responses, and cellular survival [64]. The NF-κB pathway is activated in response to AGE binding to RAGE, leading to the production of proinflammatory cytokines, chemokines, and adhesion molecules, which promote the recruitment of immune cells to the injury site [65]. NF-κB activation also increases the production of ROS, which further amplifies the inflammatory response and contributes to oxidative stress and tissue damage [66].

For example, in DN, NF-κB activation promotes the expression of cytokines such as TNF-α and IL-6, which effectively contribute to the chronic inflammation observed in the kidneys [67]. This inflammation exacerbates glomerular injury, increases vascular permeability, and promotes the accumulation of extracellular matrix proteins, leading to fibrosis and renal dysfunction [68]. In diabetic retinopathy, NF-κB activation in retinal cells contributes to the breakdown of the blood–retinal barrier, promoting retinal edema and the progression of vision impairment [63]. Furthermore, NF-κB signaling plays a role in the development of insulin resistance by inducing the expression of inflammatory mediators that impair insulin receptor signaling [69]. The role of NF-κB in AGE-induced inflammation underscores the importance of this transcription factor as a therapeutic target for mitigating diabetic complications and slowing the progression of the disease.

### 4.4. AGEs and the PI3K/Akt Pathway

The PI3K/Akt pathway is a central regulator of cellular metabolism, growth, and survival, playing a crucial role in maintaining insulin sensitivity [70]. In the context of diabetes, AGE accumulation activates PI3K/Akt pathway upon AGEs binding to the RAGE, leading to the phosphorylation of Akt, a protein kinase that regulates numerous cellular processes, including glucose metabolism, cell survival, and protein synthesis [71]. However, in diabetic conditions, excessive activation of this pathway can promote insulin resistance and impaired glucose uptake in peripheral tissues [72].

In muscles, the PI3K/Akt pathway plays a particularly crucial role in regulating endothelial cell function and maintaining vascular homeostasis [73]. AGE-induced activation of PI3K/Akt leads to the disruption of endothelial cell signaling, contributing to endothelial dysfunction, vascular permeability, and the development of atherosclerosis [74]. In pancreatic β-cells, the PI3K/Akt pathway is also involved in regulating insulin secretion and β-cell survival [75]. Further AGE-induced dysregulation of this pathway impairs insulin secretion and promotes β-cell apoptosis, further contributing to the progression of T2D [76].

### 4.5. NLRP3 Inflammasome Activation

The NLRP3 inflammasomes comprise the NLRP3 sensor, ASC adaptor, and pro-caspase-1, that is a critical mediator of AGE-induced inflammatory signaling [77]. With reference to diabetes, NLRP3 is activated downstream of the AGE–RAGE axis and plays a fundamental role in promotion of chronic low-grade inflammation and tissue injury. Engagement of RAGE by AGEs initiates oxidative stress and NF-κB activation, which prime the transcription of NLRP3 and its downstream cytokines, pro-IL-1β and pro-IL-18 [78]. Subsequent AGE-induced mitochondrial dysfunction, reactive oxygen species (ROS) overproduction, and ion flux imbalances serve as activation signals that promote assembly of the NLRP3 inflammasome complex. The activation of caspase-1 within this complex leads to the maturation and secretion of IL-1β and IL-18, as well as pyroptotic cell death [79], thereby amplifying inflammatory responses and contributing to the progression of diabetic complications.

### 4.6. PKC Pathway

In diabetes, continued hyperglycemia upsurges the intracellular diacylglycerol (DAG) levels, which unreasonably activate various protein kinase C (PKC) isoforms, particularly PKC-β, PKC-δ, and PKC-θ [80]. The PKC activation alters vascular and metabolic homeostasis by stimulating NADPH oxidase–dependent reactive oxygen species (ROS) production, activating NF-κB and MAPK pathways, and finally spoils the insulin receptor signaling [81]. In vascular tissues, PKC promotes endothelial dysfunction by reducing nitric oxide (NO) bioavailability thereby increasing endothelin-1 expression and enhancing vascular permeability. In renal cells, PKC signaling accelerates mesangial expansion, extracellular matrix deposition, and glomerular sclerosis that finally leads to diabetic nephropathy [82]. Similarly in retina, PKC-β activation drives VEGF overexpression, neovascularization, and capillary leakage, exacerbating diabetic retinopathy [83]. Moreover, PKC cross-talks with the AGE–RAGE pathway by amplifying oxidative stress and inflammation, creating a synergistic loop that accelerates microvascular complications [84].

### 4.7. Other Signaling Pathways Involved in AGE-Induced Diabetic Complications

In addition to these well-established pathways, the role of several other signaling pathways is also important for consideration. One such pathway is the TGF-β/Smad signaling pathway, which plays a critical role in tissue fibrosis and remodeling [85]. The AGE accumulation activates TGF-β signaling, leading to the production of extracellular matrix components, fibrosis, and renal dysfunction [86]. The activation of TGF-β signaling by AGEs can occur through both direct (AGE binding to RAGE) and indirect mechanisms (activation of oxidative stress and inflammation). TGF-β also promotes the differentiation of fibroblasts into myofibroblasts, which contribute to tissue scarring and organ damage. Additionally, AGEs can modulate autophagy pathways, particularly in pancreatic β-cells, where impaired autophagy contributes to β-cell dysfunction and insulin secretion deficits [87]. AGEs inhibit autophagic processes in pancreatic β-cells, leading to the accumulation of damaged proteins and organelles, further impairing β-cell function. This interference is mainly through RAGE–NF-κB pathway. The AGEs to RAGE attachment activates NF-κB signaling, which in turn suppresses the expression of key autophagy-related genes, including Beclin-1 and LC3 [88]. These additional pathways, along with those already discussed, contribute to the multi-organ complications observed in diabetes and underscore the complexity of AGE-mediated pathophysiology (Table 2).

## 5. Phenolic Compounds in Diabetes Management

Phenolic compounds, a diverse group of naturally occurring phytochemicals, have gained significant attention for their potential therapeutic effects in managing diabetes and any associated complications [96]. Phenolic compounds include flavonoids, phenolic acids, stilbenes, and lignans, and each possesses unique biological properties [97]. These compounds have been extensively studied for their antioxidant, anti-inflammatory, anti-glycation, and anti-hyperglycemic effects, all of which play a key role in modulating the underlying mechanisms of diabetes [90] and reducing the associated risks.

### 5.1. Major Dietary Sources of Phenolic Compounds

Phenolic compounds are widely distributed in plant-based foods, and the consumption of these compounds has been linked to various health benefits, particularly in managing several ailments. These compounds are present in a diverse range of fruits, vegetables, herbs, and spices, which are staples in many diets worldwide [98]. Flavonoids, phenolic acids, stilbenes, and lignans are the primary classes of phenolic compounds found in food sources [99].

As presented earlier, numerous dietary sources of phenolic compounds have been reported, including berries, vegetables, herbs, and fruits. Indeed, berries, such as blueberries, strawberries, and raspberries, are rich in flavonoids, including anthocyanins, which have been shown to promote potent antioxidant, anti-AGE, and anti-inflammatory effects [100]. These food sources are also high in vitamin C, which further enhances their ability to scavenge ROS and reduce oxidative stress [101]. Citrus fruits, such as oranges, lemons, and grapefruits, are another excellent source of flavonoids, particularly flavanones, such as hesperidin and naringenin, which have been linked to improved insulin sensitivity and glucose metabolism [102]. Additionally, the consumption of apples has been associated with reduced inflammation and oxidative stress [103], owing to the rich content of phenolic acids in apples, particularly chlorogenic acid, which is known to inhibit AGE formation and reduce hyperglycemia [104].

Vegetables are another important source of phenolic compounds. Cruciferous vegetables, such as broccoli, brussels sprouts, and kale, contain high levels of phenolic acids, particularly ferulic acid, which has been shown to exhibit strong antioxidant and anti-inflammatory properties [105]. Other vegetables, such as spinach, onions, and tomatoes, are rich in flavonoids and other phenolic compounds that contribute to improved metabolic health and reduced inflammation [106]. Meanwhile, the consumption of herbs and spices, such as turmeric, ginger, cinnamon, and oregano, also provides a significant amount of phenolic compounds with various biological activities [107]. For example, curcumin, the active compound in turmeric, has been extensively studied for its anti-inflammatory and anti-glycation effects, making this spice a valuable dietary supplement in diabetes management [108]. Green tea represents another prominent source of phenolic compounds, particularly catechins, which have been shown to improve glucose metabolism, reduce oxidative stress, and enhance insulin sensitivity [109]. The broad spectrum of phenolic compounds present in these food groups supports their potential role in managing diabetes and its complications through dietary interventions.

### 5.2. Mechanisms of Action of Phenolic Compounds

The mechanisms through which phenolic compounds exert their effects in diabetes management are multifaceted. The antioxidant properties of phenolic compounds are crucial to their ability to combat oxidative stress, a hallmark of diabetes. By scavenging ROS, phenolic compounds protect cellular components from oxidative damage and limit the formation of AGEs, which are implicated in various diabetic complications [110]. ROS are produced as byproducts of normal metabolic processes, but their levels can become elevated under pathological conditions, such as hyperglycemia. These elevated ROS levels lead to cellular dysfunction by damaging proteins, lipids, and nucleic acids, triggering inflammatory responses, and promoting the formation of AGEs [111]. The antioxidant effects of phenolic compounds neutralize ROS, thereby reducing the cascade of events that cause cellular damage and the progression of diabetic complications [112]. Among these known phenolic compounds, flavonoids such as quercetin, catechins, and resveratrol have been shown to possess potent antioxidant activity [113], making these compounds promising candidates for diabetes management.

In addition to their antioxidant effects, phenolic compounds exhibit strong anti-inflammatory properties that contribute to their therapeutic potential in diabetes. Chronic inflammation is a characteristic feature of diabetes, particularly in adipose tissue, skeletal muscle, and the vasculature [114]. Meanwhile, chronic inflammation is driven by elevated levels of proinflammatory cytokines such as TNF-α, IL-6, and IL-1β, which contribute to insulin resistance and endothelial dysfunction [115]. Phenolic compounds modulate the expression of these cytokines by inhibiting the activation of transcription factors such as NF-κB, which regulates the expression of many inflammatory genes [116]. For instance, resveratrol, a stilbene found in grapes and red wine, has been shown to inhibit NF-κB activation and reduce the production of proinflammatory cytokines in vitro and in vivo [117]. Other phenolic compounds, such as curcumin and catechins, also exhibit anti-inflammatory effects by downregulating the expression of inflammatory mediators, contributing to improved insulin sensitivity and reduced risk of diabetic complications. Furthermore, phenolic compounds have been shown to inhibit enzymes involved in carbohydrate digestion, such as α-amylase and α-glucosidase, thereby slowing glucose absorption and improving postprandial glycemic control [118].

### 5.3. Reducing AGE Formation

One of the most promising therapeutic actions of phenolic compounds in diabetes management is their ability to inhibit the formation of AGEs. As discussed earlier, AGEs play a pivotal role in the pathogenesis of diabetic complications, including nephropathy, retinopathy, and cardiovascular disease. However, several phenolic compounds have been shown to inhibit the Maillard reaction through which AGEs are produced, thereby reducing the formation of AGEs [119]. This effect is particularly important in the context of diabetes, where excessive AGE formation contributes to tissue damage and organ dysfunction [120]. Phenolic compounds, such as chlorogenic acid, curcumin, and resveratrol, have also demonstrated an ability to reduce AGE formation not only by directly inhibiting the glycation of proteins but also through scavenging the reactive intermediates [121]. Chlorogenic acid, a phenolic acid found in coffee and certain fruits, has also been shown to reduce AGE formation in vitro by inhibiting the interaction between glucose and proteins [122]. Similarly, curcumin has been reported to inhibit the formation of AGEs and reduce the accumulation of AGEs in diabetic tissues [123]. Likewise, resveratrol possesses anti-glycation properties and has been shown to reduce the formation of AGEs in both in vitro and in vivo models of diabetes [124].

#### 5.3.1. Inhibition of Free Radical Formation

ROS are generated during the early stages of glycation and can further promote the formation of AGEs. Phenolic compounds possess antioxidant properties that enable the compounds to scavenge these free radicals, thereby reducing oxidative stress and limiting the subsequent formation of AGEs [125,126].

#### 5.3.2. Scavenging of Reactive Dicarbonyl Compounds

MGO and dicarbonyl compounds are extremely reactive intermediates in the glycation pathway in DM [127]. Phenolic compounds can trap these intermediates through nucleophilic attack, forming stable adducts that prevent the progression to AGEs [128].

#### 5.3.3. Metal Ion Chelation

Transition metals, such as iron (Fe^2+^) and copper (Cu^2+^), catalyze the oxidation of Amadori products, facilitating the formation of AGEs [129]. Phenolic compounds can chelate these metal ions, reducing their availability and, thus, inhibiting metal-catalyzed oxidative reactions that lead to AGE formation [130].

#### 5.3.4. Modulation of Glyoxalase I Activity

The glyoxalase system, particularly glyoxalase I (GLO I), also plays a crucial role in detoxifying MGO by converting it into less reactive compounds [131]. Certain phenolic compounds, including quercetin, curcumin and resveratrol etc., [132,133] can enhance the activity of GLO I, thereby increasing the clearance of MGO and reducing the formation of AGEs [134].

#### 5.3.5. Inhibition of AGE–Receptor Interactions

**Figure 3:**
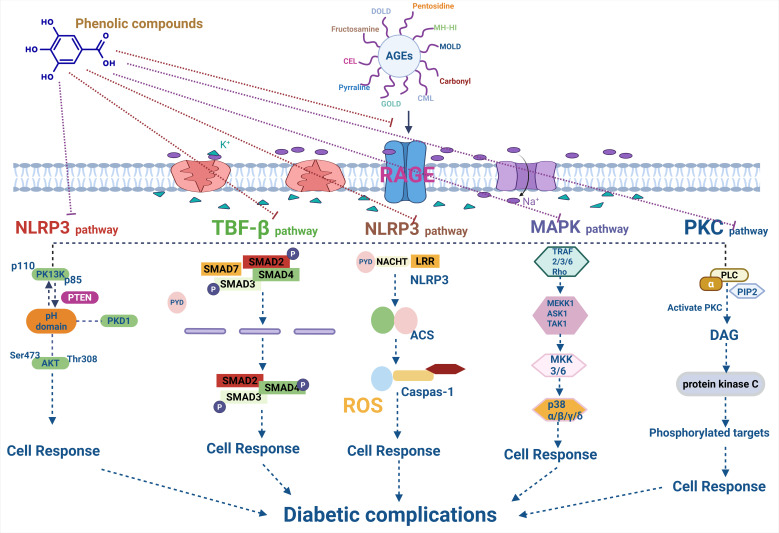
Mechanistic insights of phenolic compounds in AGEs inhibition and Diabetic complications.

AGEs exert their pathological effects by binding to the RAGE, triggering the inflammatory and oxidative pathways. Phenolic compounds can inhibit the expression of the RAGE or block its interaction with AGEs, thereby mitigating the downstream inflammatory responses associated with AGE accumulation [135] (Figure 3 and Table 3).

### 5.4. Pharmacokinetics of Phenolic Compounds

Despite the promising therapeutic effects of phenolic compounds, the clinical application of these compounds has remained limited due to their relatively poor bioavailability [148]. Notably, many phenolic compounds are poorly absorbed in the gastrointestinal tract due to their low solubility and rapid metabolism [149]. Therefore, only a small fraction of the phenolic compounds consumed through dietary methods reaches systemic circulation in an active form, thereby limiting their therapeutic efficacy. Several factors influence the bioavailability of phenolic compounds, including their molecular size, solubility, and stability in the digestive system [150]. Additionally, phenolic compounds undergo extensive first-pass metabolism in the liver, where these compounds are converted into metabolites with potentially reduced biological activity [151].

Therefore, various strategies have been employed to enhance the bioavailability of phenolic compounds, including the use of advanced drug delivery systems, such as nanoparticles, liposomes, and micelles [30]. These delivery systems can encapsulate phenolic compounds, improving their solubility, stability, and absorption. Nanoparticles, for example, can protect phenolic compounds from degradation in the gastrointestinal tract and facilitate their targeted delivery to specific tissues, such as the liver, kidneys, or adipose tissue [152]. Another approach to improving the bioavailability of phenolics involves modifying the chemical structure of these compounds to increase solubility and stability [28]. For instance, esterification of phenolic acids can enhance lipophilicity and improve absorption in the intestines [153]. Additionally, combining phenolic compounds with other bioactive agents, such as lipids or essential oils, can enhance their pharmacokinetic properties and therapeutic efficacy [154]. These strategies are critical for maximizing the potential of phenolic compounds as therapeutic agents in the management of diabetes and any associated complications.

## 6. Evidence from Experimental Studies on Phenolic Compounds in Diabetes

Numerous experimental studies have been conducted to evaluate the therapeutic potential of phenolic compounds in managing diabetes and its complications. Indeed, these in vitro studies, animal models, and human clinical trials have provided valuable insights into the biological mechanisms through which phenolic compounds exert their effects, particularly in reducing oxidative stress, inflammation, and AGE formation. These compounds have shown promising results in preventing and mitigating diabetic complications, including DN, retinopathy, neuropathy, and cardiovascular disease. The accumulation of evidence from these studies suggests that phenolic compounds can play an integral role in diabetes management by targeting multiple pathways involved in the pathophysiology of the disease.

### 6.1. In Vitro Studies

In vitro studies have played a pivotal role in demonstrating the antioxidant, anti-inflammatory, and anti-glycation effects of phenolic compounds. These studies typically involve the use of cultured cells exposed to diabetic conditions, such as high glucose levels or AGEs, to assess the impact of phenolic compounds on cellular function. One of the key findings from these in vitro studies is that phenolic compounds, such as quercetin, resveratrol, and curcumin, possess potent antioxidant activity that helps neutralize ROS and prevent oxidative stress [155,156,157].

Similarly, in diabetic cell models, phenolic compounds, such as gallic acid, epigallocatechin-3-gallate, resveratrol, oleuropein, and curcumin, have been shown to reduce ROS-mediated oxidative damage to lipids, proteins, and DNA, thereby protecting cells from the deleterious effects of hyperglycemia [112,158]. ROS include both oxygen-centered radicals, such as O_2_^-^ or hydroxyl (OH^•^) radicals, and other non-radical oxygen derivatives, including hydrogen peroxide (H_2_O_2_) and singlet oxygen (^1^O_2_) [159]. Additionally, phenolic compounds, including punicalagin (PC), ellagic acid, naringin, hesperidin, and rutin, have been found to modulate the expression of proinflammatory cytokines, such as TNF-α, IL-6, and IL-1β, which are elevated in diabetes and contribute to insulin resistance [160,161]. Likewise, isorhamnetin, naringenin, and pelargonidin have been shown to inhibit NF-κB activation in macrophages, reducing the production of inflammatory cytokines and improving insulin sensitivity [162].

Phenolic compounds are also known to exert anti-glycation effects that are critical in reducing AGE formation. Several studies have demonstrated that compounds such as chlorogenic acid, curcumin, and catechins can directly inhibit the Maillard reaction, preventing the glycation of proteins and the subsequent formation of AGEs [21,163]. In vitro studies using endothelial cells, for example, have demonstrated that homoprotocatechuic and ferulic acids, as well as quercetin, can inhibit the accumulation of AGEs, thereby reducing AGE-mediated cellular dysfunction and improving endothelial function [164,165]. Thus, by targeting the pathways involved in AGE formation and reducing oxidative stress, phenolic compounds hold significant potential as potential therapeutic agents for managing diabetic complications, as evaluated in vitro

### 6.2. In Vivo Studies

In vivo studies in animal models of diabetes have provided further evidence supporting the therapeutic efficacy of phenolic compounds in preventing and treating diabetic complications. These studies are particularly valuable in assessing the overall impact of phenolic compounds on glucose metabolism, insulin resistance, and tissue damage in a living organism. Animal studies using diabetic models have shown that phenolic compounds, such as epigallocatechin-3-O-gallate (EGCG) from green tea, can significantly inhibit AGEs and thereby play a role in the management of diabetic complications [166]. Similarly, animal studies have explained the role of resveratrol and curcumin in significantly lowering hyperglycemia, inhibiting AGEs, and playing a role in mitigating the effects of neurodegenerative diseases (NDDs) [121,167].

The effects of phenolic compounds on diabetic complications have also been demonstrated in animal models of DN, retinopathy, and cardiovascular disease. Curcumin has been shown to reduce kidney damage in diabetic rats by decreasing proteinuria, inflammation, and fibrosis [168]. Moreover, the ability of curcumin to modulate the AGE–RAGE signaling pathway and reduce oxidative stress has been suggested as the underlying mechanism of its renoprotective effects [169]. Meanwhile, phenolic compounds such as gallic acid, protocatechuic acid, *p*-hydroxybenzoic acid, *p*-coumaric acid, vanillic acid, and quercetin have been shown to protect retinal cells from AGE-induced damage, reduce vascular leakage, and prevent retinal edema in animal models of diabetic retinopathy [170,171]. Furthermore, stilbenes, including trans- and cis-resveratrol, piceatannol, and viniferins, have demonstrated cardioprotective effects by improving endothelial function, reducing arterial stiffness, and preventing the progression of atherosclerosis in diabetic animal models [172]. These studies provide strong evidence that phenolic compounds can prevent or delay the onset of diabetic complications and improve overall metabolic health in vivo.

### 6.3. Human Clinical Trials and Observational Studies

Human clinical trials and observational studies have provided additional insights into the effectiveness of polyphenolic compounds in managing diabetes and its complications. While in vitro and animal studies provide important mechanistic data, clinical trials are crucial for assessing the real-world applicability and safety of phenolic compounds in humans. Several clinical trials have evaluated the effects of phenolic-rich dietary interventions, such as the consumption of berries, green tea, and red wine, on glycemic control and AGE levels in diabetic patients [173,174,175]. One notable study demonstrated that the intake of polyphenol-rich foods, such as blueberries and strawberries, significantly improved insulin sensitivity and reduced fasting blood glucose levels in individuals with T2D [176]. These findings suggest that dietary phenolic compounds can have a beneficial impact on glycemic control and may complement conventional diabetes treatments.

In addition to improving glucose metabolism, clinical studies have also demonstrated that phenolic compounds can reduce inflammation and oxidative stress biomarkers in patients with DM. For example, resveratrol supplementation was found to decrease serum levels of proinflammatory cytokines and reduce markers of oxidative damage in patients with T2D [177]. Moreover, studies have suggested that diets rich in polyphenols, including hesperidin and resveratrol, are associated with a lower risk of developing diabetic complications, as these compounds can influence various factors related to AGE production [178]. The clinical evidence supports the notion that phenolic compounds can help manage diabetes and its associated complications through their antioxidant, anti-inflammatory, and anti-glycation properties [179]. However, further well-designed large-scale clinical trials are needed to confirm these findings and establish the optimal dosages and long-term effects of phenolic compounds in diabetes management. Similarly, various clinical investigations based on polyphenols from the whortleberry, olive oil, coffee, guava tea, propolis, red wine, grape seed, and cocoa have shown that these are very effective in showing antidiabetic effects in patients with T2D through increasing glucose metabolism and improving vascular function, as well as reducing insulin resistance and HbA1c levels [180].

Though numerous human clinical trials and observational studies have assessed the effects of phenolic-rich dietary interventions, such as the consumption of berries, green tea, and red wine, on glycemic control and metabolic health, it is important to note that these trials do not directly assess pure or combined phenolic compounds. Furthermore, AGEs were not measured as outcomes in these studies, which limits our ability to draw definitive conclusions regarding the direct impact of phenolic compounds on AGE formation in humans. This limitation is acknowledged, and further research that directly evaluates phenolic compounds and quantifies AGEs as an outcome measure is needed to better understand the potential therapeutic role of phenolics in managing diabetic complications.

### 6.4. The Toxicity of Polyphenolic Compounds and Their Interactions

Despite extensive biological activities of polyphenolic compounds, there exist a great of uncertain and side effects too [181]. It has been reported earlier in several investigations that intake of higher amounts of dietary polyphenols or excessive use of supplements can lead to undesirable consequences, including G.I.T. disturbances, impaired nutrient absorption and metal ion chelation [182,183]. For instance, strong iron-binding activity has been associated with reduced bioavailability of this essential micronutrient, raising concerns for populations with marginal iron status. More importantly, the biological effects of polyphenols are dose- and form-dependent, with purified aglycones or concentrated extracts often produce unusual effects that are not commonly observed with polyphenols consumed naturally within whole foods [184]. Similarly, polyphenol–drug interactions and the occurrence of potential pro-oxidant activities is another important concern to consider [185]. Polyphenols are capable of modifying activity of cytochrome P450 enzymes in liver and various drug transporters, that can alter the pharmacokinetics of co-administered medicines thereby reducing therapeutic efficacy of drugs or enhancing their toxicity [186]. Additionally, although polyphenols are primarily observed as antioxidants, some can exhibit pro-oxidant properties at high concentrations, generating reactive oxygen species that induce genotoxic or mutagenic effects in experimental systems [187]. Polyphenols can also influence hormonal signaling pathways, with implications for endocrine balance. Overall there exists a great need for caution in polyphenol supplement use and the importance of further studies on safety thresholds and interactions.

## 7. Modern Computational Tools in AGE and Phenolic Compounds Research

Modern computational tools play a crucial role in advancing our understanding of the molecular mechanisms underlying the effects of AGEs and phenolic compounds in diabetes management. These tools enable researchers to study the interactions between AGEs and their receptors, such as the RAGE, as well as the binding affinity and efficacy of phenolic compounds in inhibiting AGE formation and modulating key cellular pathways [188]. By integrating molecular docking, molecular dynamics (MD) simulations, and network pharmacology approaches, researchers can gain insights into the structure–activity relationships of phenolic compounds and their potential therapeutic benefits [189,190].

### 7.1. Molecular Docking and Virtual Screening

Molecular docking is a computational technique used to predict the binding interactions between small molecules, such as phenolic compounds, and their target proteins, including those involved in AGE formation or the AGE–RAGE signaling pathway [191]. Docking simulations provide valuable information regarding the binding affinity, orientation, and binding sites of compounds with specific receptors or enzymes [192], helping to identify the most promising candidates for therapeutic intervention. This approach has been widely employed to study the interactions between AGEs and RAGE, as well as to explore how phenolic compounds can disrupt these interactions. For instance, docking studies have demonstrated that polyphenolic compounds, such as resveratrol and quercetin, can bind to the RAGE with high affinity, thereby inhibiting the AGE–RAGE interaction and preventing AGE-induced inflammatory responses [193,194]. Such findings support the potential of phenolic compounds as therapeutic agents for mitigating AGE-related complications in diabetes. Similarly, citrus flavonoids, including hesperidin and hesperetin, have been analyzed through in vitro and docking studies to investigate their interaction with the AGE/RAGE/NF-κB pathway, yielding significant results [195].

### 7.2. Molecular Dynamics Simulations

MD simulations offer a powerful method for studying the time-dependent behavior of molecular systems [196], providing detailed insights into the dynamic interactions between phenolic compounds, AGEs, and their receptors. Unlike static docking studies, MD simulations capture the flexibility of molecules and the conformational changes that may occur during binding [197]. This enables researchers to investigate the stability of the AGE–RAGE complex and to assess the effect of phenolic compounds on the structural integrity and function of the receptor. MD simulations have been used to investigate the interactions between RAGE and AGEs, revealing the mechanisms through which AGE binding induces conformational changes that facilitate the activation of downstream signaling pathways. MD simulations can also provide valuable insights into the binding dynamics of phenolic compounds with enzymes involved in AGE formation, such as GLO I and α-amylase [198]. By simulating the binding of phenolic compounds to these enzymes, researchers can assess the ability of these compounds to inhibit enzyme activity and, in turn, reduce the production of AGEs. For example, MD simulations have shown that certain flavonoids, such as epicatechin and quercetin, can effectively inhibit GLO I, thereby preventing the accumulation of reactive di-carbonyl compounds that contribute to AGE formation [199]. These simulations offer a detailed understanding of the molecular mechanisms through which phenolic compounds exert anti-glycation effects, providing a solid foundation for the design of more effective AGE-targeting therapeutics. In another investigation, MD simulations were performed to cover flavonoids with the RAGE. The MD simulation confirmed that flavonoids, including icariin, kaempferol, luteolin, and quercetin, formed stable complexes with the RAGE. The study identified the RAGE as a novel therapeutic target for epimedium in mitigating VaD through its anti-inflammatory properties [200]. Likewise, MD investigations on glycolipid metabolic disorders such as nonalcoholic fatty liver disease (NAFLD), obesity, and DN have been found to show a significant interaction of dietary polyphenols with their receptors [201].

### 7.3. Network Pharmacology Approaches

Network pharmacology is an emerging computational approach that integrates systems biology and pharmacology to understand the complex interactions between drugs, targets, and biological pathways [202]. In the context of AGEs and phenolic compounds, network pharmacology approaches can be effectively used to identify the key molecular targets involved in AGE formation, AGE–RAGE signaling, and diabetic complications. This approach allows researchers to construct molecular networks that highlight the interconnected pathways and potential therapeutic targets that phenolic compounds can modulate [203]. Thus, by mapping these interactions between phenolic compounds and the proteins involved in AGE-related pathways, network pharmacology can provide a comprehensive view of the mechanisms through which these compounds exert their therapeutic effects across multiple biological systems [204]. In the context of AGEs and phenolic compounds, one of the primary applications of network pharmacology in the research of AGEs and phenolic compounds is the identification of multi-target effects [205]. Unlike traditional drug discovery methods that focus on single-target molecules, network pharmacology recognizes that diseases, such as diabetes, often involve complex, multifaceted pathways. Several investigators have successfully explored the network pharmacology of phenolic compounds and AGEs. For example, network pharmacology studies have revealed that resveratrol inhibits AGE formation and modulates inflammation, oxidative stress, and insulin resistance, thereby making resveratrol a promising multi-target therapeutic agent [206]. Similarly, network pharmacology analyses have confirmed that resveratrol significantly inhibits intestinal aging by downregulating the ATF4/Chop/Bcl-2/Bax signaling pathway, a finding further confirmed in animal studies [207]. Similarly, the use of network pharmacology in AGE and phenolic compound research can also facilitate the identification of novel targets for drug development and the optimization of existing therapeutic strategies. For instance, an investigation on Gegen Qinlian (GQL) (mainly comprising puerarin, baicalin, and wogonin) decoction confirmed through network pharmacology that the antidiabetic effects of GQL were associated with modulating the TNF and PI3K–AKT–MTOR pathways. These findings were further supported by in vivo experimental data [208].

Presently, strategies to integrate data from molecular docking, MD simulations, network pharmacology, and experimental studies are commonly employed to simulate the effects of compounds on cellular systems and organs [209]. These models are equally valuable in AGE and phenolic interactions analyses [210,211].

### 7.4. Bioinformatics Tools for Gene Expression and Pathway Analyses

Bioinformatics tools for gene expression and pathway analyses are instrumental in understanding the molecular mechanisms underlying various health complications and the effects of drug moieties on these pathways [212,213]. These tools can also be efficiently used for AGEs. For example, gene expression analysis allows researchers to identify changes in the expression of genes involved in AGE formation, inflammation, oxidative stress, and insulin resistance in response to treatment with phenolic compounds [214,215]. High-throughput technologies, such as RNA sequencing (RNA-seq), enable the identification of differentially expressed genes (DEGs), which can then be mapped to relevant signaling pathways using bioinformatics tools, including Kyoto Encyclopedia of Genes and Genomes (KEGG), Reactome, and Gene Ontology (GO) [215] Specifically, several polyphenolic compounds such as resveratrol, quercetin, and curcumin have been analyzed using bioinformatic tools. For instance, resveratrol was evaluated using molecular docking studies to assess its binding affinity to the receptor for advanced glycation end products (RAGE) [193], while quercetin and curcumin were analyzed for their ability to modulate inflammatory pathways through interactions with NF-κB and MAPK signaling [216].

**Figure 4:**
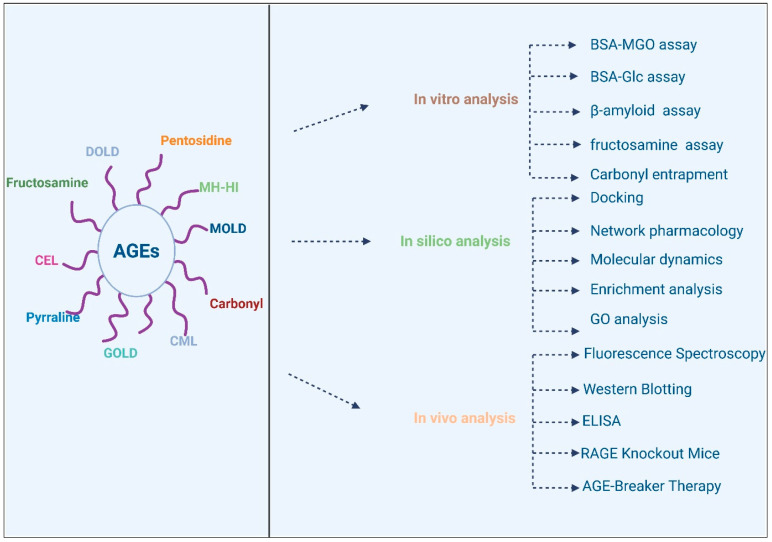
Analysis of AGEs using various models.

Pathway enrichment analysis is another key tool in bioinformatics that helps researchers understand the biological significance of DEGs and their involvement in specific pathways [217]. By applying pathway analysis tools, researchers can identify the key signaling networks affected by phenolic compounds, such as the AGE–RAGE pathway, NF-κB signaling, and insulin resistance pathways [218]. This approach allows for the identification of novel biomarkers and therapeutic targets, as well as a deeper understanding of how phenolic compounds can modulate complex biological processes to mitigate diabetic complications. Various investigations have previously utilized these tools, and key outcomes have been reported in relation to phenolic compounds and the enrichment analysis of the AGE pathway [218]. Figure 4 provides a comprehensive overview of the various analysis tools that are used to investigate various AGES types, including GOLD, MOLD, CML, carbonyl groups, etc. The AGES research has been broadly divided into in silico analysis, in vitro analysis and in vivo analysis. initially, various predictions regarding bioavailability, ligand-target interaction, and Molecular dynamics can be performed to forecast the potential of the tested molecule for further research. This is further assisted by in silico models to validate the outcomes of in silico analysis. Finally, once both these analysis models are aligned, in vivo analysis is performed to validate all outcomes.

## 8. Therapeutic Potential and Future Directions

The therapeutic potential of polyphenolic compounds in diabetes management has garnered significant attention due to their multifaceted biological properties, including antioxidant, anti-inflammatory, anti-glycation, and anti-hyperglycemic effects [219]. The ability of the compounds to modulate key pathological processes such as oxidative stress, inflammation, and the formation of AGEs positions them as valuable adjuncts in diabetes therapy [220]. Furthermore, phenolic compounds are known to enhance insulin sensitivity, reduce blood glucose levels, and protect against complications such as DN, retinopathy, and cardiovascular disease [221].

### 8.1. Future Research

While the therapeutic potential of polyphenolic compounds in diabetes management is well-established, several critical areas require further research to maximize their effectiveness and clinical applicability. One of the main challenges is the limited bioavailability of polyphenolic compounds, which often hinders the ability of these compounds to reach therapeutic concentrations in the bloodstream and target tissues [222]. The development of novel formulation strategies, including nanoparticles, liposomes, or other drug delivery systems, can help enhance the bioavailability of phenolic compounds [223].

In addition to improving bioavailability, more studies are needed to investigate the optimal doses, durations, and combinations of polyphenolic compounds for managing diabetes [224]. While several clinical trials have demonstrated the benefits of phenolic-rich diets or supplements in improving glycemic control and reducing diabetic complications, limited information remains on the long-term effects of these compounds [225]. Therefore, future studies should focus on conducting large-scale, well-designed clinical trials that evaluate the safety and efficacy of phenolic compounds over extended periods, particularly in diverse populations with different forms of diabetes [219,226]. Moreover, the potential for synergistic effects between phenolic compounds and conventional diabetes medications should be explored, as combining natural and pharmaceutical therapies could enhance treatment outcomes and provide a more holistic approach to managing diabetes [227].

### 8.2. Potential Synergism with Antidiabetic Drugs

The combination of polyphenolic compounds with conventional antidiabetic drugs has shown great promise in improving diabetes management. The polyphenolic compounds, such as resveratrol, quercetin, and curcumin, possess antioxidant, anti-inflammatory, and anti-glycation properties that can complement the action of conventional antidiabetic drugs. For instance, metformin, the first-line therapy for T2D, functions by improving insulin sensitivity and reducing hepatic glucose production [228]. The polyphenolic compound resveratrol has been shown to enhance insulin sensitivity by modulating pathways such as AMP-activated protein kinase (AMPK) [229], which is also activated by metformin. The combined action of metformin and resveratrol can lead to enhanced glucose uptake in muscle cells and improved glucose homeostasis. Similarly, polyphenolic compounds can complement sulfonylureas, which stimulate insulin secretion from pancreatic β-cells [230]. Hence, by reducing oxidative stress and inflammation, polyphenolic compounds improve β-cell function and insulin secretion, enhancing the effectiveness of sulfonylureas and potentially decreasing the required doses.

## 9. Conclusions

The polyphenolic compounds derived from numerous plant-based foods offer significant therapeutic potential in managing diabetes and any subsequent complications. polyphenolic compounds possess potent antioxidant, anti-inflammatory, anti-glycation, and anti-hyperglycemic properties, which provide a multifaceted approach to addressing the pathophysiology of diabetes. By modulating key biological processes such as oxidative stress, inflammation, and the formation of AGEs, polyphenolic compounds play a crucial role in reducing the risk and severity of diabetic complications, including nephropathy, retinopathy, and cardiovascular disease. Additionally, the ability of these compounds to enhance insulin sensitivity, regulate blood glucose levels, and improve overall metabolic health further underscores the promising role of phenolic compounds in diabetes therapy. The accumulation of evidence from in vitro, in vivo, and clinical studies highlights the effectiveness of phenolic compounds in enhancing glucose metabolism, mitigating oxidative stress, and counteracting the detrimental effects of AGEs. These compounds, such as resveratrol, curcumin, and quercetin, have demonstrated beneficial effects on insulin signaling, AGE–RAGE signaling, and efficient glycemic control, making phenolics valuable adjuncts to conventional diabetes treatments. Moreover, the anti-inflammatory effects of polyphenolic compounds help alleviate the chronic low-grade inflammation that contributes to the progression of diabetes and any subsequent complications. However, despite the promising biological activities of these compounds, the clinical application of polyphenolic compounds is hindered by challenges related to their bioavailability. The low solubility, rapid metabolism, and limited absorption of these compounds in the gastrointestinal tract reduce their efficacy at therapeutic doses. Therefore, addressing these bioavailability challenges through innovative formulation strategies, such as nanoparticle delivery systems, is essential for enhancing the therapeutic potential of polyphenolic compounds. Furthermore, additional research, particularly large-scale clinical trials, is necessary to establish optimal dosages, long-term safety, and efficacy in diverse diabetic populations.

As the body of research continues to grow, polyphenolic compounds hold great promise not only as therapeutic agents in the management of diabetes but also as preventative measures to reduce the risk of disease progression in high-risk individuals. The natural origin of these compounds, combined with their potential to target multiple pathways involved in diabetes pathogenesis, makes them an attractive option for inclusion in diabetes care strategies. Ongoing advancements in formulation techniques and clinical research may make polyphenolic compounds an integral part of diabetes management, providing patients with a complementary and effective treatment option.

## Figures and Tables

**Table 1:** Key Studies on Phenolic Compounds and AGEs in Diabetes Management.

S.No	Phenolic Compound	Diabetes Complication Addressed	No Key Findings	Methodology
1	Quercetin	Diabetic Nephropathy	Prevents AGE accumulation, reduces oxidative stress, modulates RAGE signaling, enhances renal function	In vitro, Animal Model
2	Resveratrol	Diabetic Retinopathy	Reduces retinal edema, prevents vascular leakage, inhibits AGE formation, modulates AGE-RAGE signaling	In vivo, Animal Model, Clinical Trial
3	Curcumin	Diabetic Nephropathy	Inhibits AGE formation, reduces kidney damage, enhances antioxidant defenses, modulates RAGE signaling	In vivo, Animal Model, Clinical Trial
4	Chlorogenic Acid	Diabetic Complications	Inhibits glycation, reduces AGE formation, improves glycemic control, reduces oxidative stress	In vitro, Animal Model
5	Epigallocatechin-3-gallate (EGCG)	Diabetic Retinopathy	Inhibits AGE accumulation, reduces vascular leakage, prevents retinal damage, improves insulin sensitivity	In vivo, Clinical Trial
6	Punicalagin	Diabetic Nephropathy	Reduces oxidative stress and inflammation, protects renal function, inhibits AGE formation	In vitro, Animal Model
7	Gallic Acid	Diabetic Retinopathy, Cardiovascular Disease	Reduces ROS, inhibits AGE formation, modulates inflammatory cytokines	In vitro, Animal Model
8	Hesperidin	Diabetic Retinopathy	Reduces AGE-induced vascular damage, improves retinal function, modulates NF-κB pathway	In vitro, Animal Model
9	Naringin	Diabetic Nephropathy	Reduces AGE formation, alleviates renal damage, inhibits oxidative stress	In vivo, Animal Model
10	Curcumin	Cardiovascular Disease	Reduces oxidative stress, enhances endothelial function, inhibits AGE formation	In vivo, Clinical Trial

**Table 2:** AGE-RAGE Signaling and Its Role in Diabetic Complications.

Signaling Pathway	Concise Mechanism	Relevant Diabetic Complication	Citation
AGE/RAGE Signaling	AGEs bind to RAGE, triggering activation of NF-κB, MAPK, and PI3K/Akt, which causes inflammation, oxidative stress, and fibrosis.	Vascular damage, diabetic retinopathy, nephropathy, neuropathy, and atherosclerosis.	[89]
NF-κB Activation	AGE binding to RAGE activates NF-κB, leading to the production of proinflammatory cytokines and adhesion molecules, promoting inflammation and endothelial dysfunction.	Diabetic vascular complications, retinopathy, nephropathy, atherosclerosis.	[90]
MAPK Pathway	RAGE activation triggers MAPK signaling (ERK, JNK, p38), promoting inflammation, cell proliferation, and apoptosis, which causes vascular damage.	Diabetic vascular complications, diabetic nephropathy and neuropathy.	[91]
PI3K/Akt Pathway	PI3K/Akt signaling, when overstimulated by AGEs, leads to oxidative stress, impaired glucose metabolism, and endothelial dysfunction.	Diabetic retinopathy, nephropathy, cardiovascular disease, atherosclerosis.	[]
TGF-β Pathway	TGF-β activation induces fibrosis by promoting ECM deposition, contributing to vascular remodeling and kidney fibrosis.	Diabetic nephropathy, diabetic cardiovascular disease, fibrosis in various tissues.	[92]
Oxidative Stress and ROS Production	AGEs induce ROS production, causing oxidative damage to proteins, lipids, and DNA, promoting endothelial dysfunction and vascular complications.	Diabetic retinopathy, nephropathy, neuropathy, and atherosclerosis.	[93]
Endothelial Nitric Oxide Synthase (eNOS)	AGE-induced oxidative stress impairs eNOS activity, leading to reduced nitric oxide production and endothelial dysfunction.	Vascular complications, atherosclerosis, hypertension, diabetic retinopathy.	[94]
NLRP3 Inflammasome Activation	AGEs activate the NLRP3 inflammasome, leading to inflammation and impaired tissue healing, exacerbating neuropathy and other complications.	Delayed wound healing, diabetic neuropathy, corneal damage, retinopathy.	[95]
PKC Pathway	AGE-induced activation of PKC promotes cell proliferation and fibrosis, exacerbating vascular damage in diabetic tissues.	Diabetic nephropathy, diabetic cardiovascular disease, vascular complications.	[]

**Table 3:** The effect of various phenolic compounds on AGEs inhibition and their mechanistic insights.

Compound Class	Mechanism	Plant Source	Role in Diabetes and Advanced Glycation End Products	Mechanism	References
Flavonoids Quercetin	Anti-inflammatory: Modulates NF-κB, MAPK to reduce chronic inflammation.	Apples, onions, citrus fruits, broccoli	Improves insulin sensitivity, reduces blood sugar, and reduces oxidative stress. Inhibits AGE formation by reducing reactive carbonyl species like MGO.	Antioxidant: Scavenges free radicals to reduce oxidative stress.	[136]
	Inhibition of glycation: Reduces carbonyl stress by inhibiting MGO.				
	Insulin sensitivity improvement: Enhances insulin receptor signaling and glucose uptake.				
Flavonoids Kaempferol	Insulin sensitizer: Improves insulin signaling and glucose metabolism.	Kale, spinach, broccoli	Reduces blood glucose levels, improves insulin sensitivity, and prevents diabetic complications like neuropathy. Prevents glycation of proteins and inhibits AGE formation.	Antioxidant: Scavenges free radicals, reducing oxidative damage.	[137,138]
	Inhibition of AGE formation: Reduces carbonyl stress and protein glycation.				
Flavonoids Anthocyanins	Inhibition of AGE formation: Reduces glycation of proteins and carbonyl stress.	Blueberries, strawberries, grapes, red cabbage	Improves insulin sensitivity, lowers blood glucose, and has potent antioxidant properties. Inhibits AGE formation by neutralizing free radicals and reducing protein glycation.	Antioxidant: Scavenges ROS, reducing oxidative damage to endothelial cells.	[139,140]
	Anti-inflammatory: Modulates NF-κB and MAPK signaling to reduce inflammation.				
Phenolic Acids Caffeic Acid	Inhibition of glycation: Reduces protein glycation and carbonyl stress.	Coffee, fruits, vegetables, sunflower seeds	Reduces blood glucose and modulates insulin secretion. Inhibits AGE formation by scavenging carbonyl species and preventing oxidative modifications of proteins.	Antioxidant: Scavenges ROS and RCS (reactive carbonyl species), reducing oxidative damage.	[141]
	Inhibition of AGE formation: Prevents glycation by decreasing carbonyl stress.				
Ferulic Acid	Inhibition of AGE formation: Inhibits MGO-mediated glycation and reduces carbonyl stress.	Whole grains, rice, oats, barley, vegetables	Controls blood sugar levels, improves insulin sensitivity, and reduces inflammation. Inhibits AGE formation and prevents glycation of proteins, reducing AGE-induced complications.	Antioxidant: Scavenges free radicals, reducing oxidative stress.	[142,143]
	Anti-inflammatory: Modulates NF-κB to reduce chronic inflammation.				
Lignans Secoisolariciresinol	Inhibition of AGE formation: Blocks protein glycation and reduces carbonyl stress, lowering AGE accumulation.	Flaxseeds, sesame seeds, whole grains	Improves insulin sensitivity, reduces oxidative stress, and protects against diabetic nephropathy. Inhibits AGE formation by blocking early stages of glycation and neutralizing ROS.	Antioxidant: Reduces ROS and RCS in diabetic tissues, protecting against oxidative damage.	[144,145]
	Insulin sensitivity: Modulates insulin receptor signaling pathways, improving glucose uptake.				
Tannins Proanthocyanidins	Inhibition of AGE formation: Prevents protein glycation by reducing carbonyl stress and scavenging reactive carbonyls.	Red wine, cocoa, grapes, berries, apple skins	Reduces oxidative stress, improves blood sugar control, and has neuroprotective effects. Prevents AGE formation by reducing glycation and neutralizing free radicals.	Antioxidant: Scavenges ROS and protects endothelial cells from oxidative damage.	[146]
	Neuroprotective: Reduces inflammation in the nervous system, protecting against diabetic neuropathy.				
	Stilbenes				
Resveratrol	Inhibition of AGE formation: Blocks AGE-RAGE signaling, reducing AGE-induced oxidative stress and vascular damage.	Red wine, grapes, peanuts	Improves insulin sensitivity, reduces blood glucose levels, and has anti-inflammatory properties. Inhibits AGE formation and reduces AGE-RAGE signaling, which is key in diabetes complications.	Antioxidant: Scavenges ROS, reducing oxidative stress in tissues.	[147]
	Insulin sensitization: Enhances insulin receptor activity, improving glucose uptake and metabolism.				

## Data Availability

No new data were created or analyzed in this study. Data sharing is not applicable to this article.
